# Reversible Nephropathy Associated with Jet Fuel Exposure

**DOI:** 10.1155/2020/2932415

**Published:** 2020-07-31

**Authors:** Latif A. Salam, Khabbab Amin, Regina Cheng, Noriyuki Murakami, Clinton Brown, Lawrence Kwon

**Affiliations:** ^1^Department of Internal Medicine, Section of Hospital Medicine, State University of New York (SUNY) Downstate Health Sciences University, 450 Clarkson Avenue, Brooklyn, NY 11203, USA; ^2^Brooklyn Health Disparities Center, Division of Nephrology, State University of New York (SUNY) Downstate Health Sciences University, 450 Clarkson Avenue, Brooklyn, NY 11203, USA; ^3^Department of Internal Medicine, Division of Nephrology, State University of New York (SUNY) Downstate Health Sciences University, 450 Clarkson Avenue, Brooklyn, NY 11203, USA

## Abstract

Acute kidney injury (AKI) with progression to oliguric or anuric acute renal failure (ARF) is often related to use of well-known nephrotoxic agents including medications such as nonsteroidal anti-inflammatory drugs (NSAIDs), angiotensin-converting enzyme inhibitors (ACEis)/angiotensin II receptor blockers (ARBs), and certain classes of antibiotics. Hyperosmolar IV contrast is also a well-known nephrotoxic agent. Severe sepsis with subsequent hypotension, marked hyperglycemia, and those with difficulty accessing water or with poor oral intake can also present with acute kidney injury related to kidney hypoperfusion, dehydration, and volume depletion. In this case report, we discover and discuss the possible effects of regular and daily occupational exposure of jet fuel (a mixture of hydrocarbons) on renal function. Jet fuel is an underdescribed and not well-known nephrotoxic agent; however, its direct toxicity on kidney function appears to be reversible with removal of exposure and aggressive fluid hydration.

## 1. Introduction

Jet fuel is a common exposure amongst those working in airports and military bases. Jet fuel is composed of a mix of hydrocarbons including mostly isoparaffins, cycloparaffins, and aromatics, as well as certain additives such as alkylated phenols, dinonylnaphthylsulfonic acid, fuel system icing inhibitor (FSII) agents, biocides, and metal deactivators [1–3]. Hydrocarbon exposure has well-documented clinical effects through inhalation, ingestion, and dermal contact [4]. Hydrocarbons undergo biotransformation primarily through the liver and accumulate in lipid-rich tissues [5]. Detoxification of organic solvents within the liver produces water-soluble compounds, which are then excreted through urine or bile, making the kidneys a potential site of injury in the metabolism of hydrocarbons found in jet fuels [6]. Few studies have been conducted on the exposure of hydrocarbons and jet fuel and their potential effect on the kidney. Experimental animal studies have demonstrated jet fuel dose-related nephropathy, with histopathologic changes seen in subchronic exposures to fuel vapors most notably in male rats [7]. In humans, the renal effects associated with acute and chronic exposure is not well defined. There are reports of glomerulonephritis among subjects with chronic exposure to hydrocarbons [8]. In Saudi Arabia, there has been a case report of a young patient (without history suggestive of connective tissue disease) with work-related exposure to jet fuel by both inhalation and direct skin contact with an initial presentation of nausea, fatigue, and bilateral flank pain. He was found to have acute renal failure with electrolyte levels within normal range, hepatitis (B and C) and HIV negative serology, ANCA/ANA negative, and normal C3/C4 [9]. Histopathologic changes have been reported in humans in association with exposure to hydrocarbons. The two histopathologic changes that have been reported are acute tubular necrosis and rapidly progressive glomerulonephritis [8]. The kidney failure reported in humans was noted at acute, as well as high levels, and appears to reversible [9].

## 2. Case Report

A 50-year-old man presented to the emergency department (ED) at SUNY Downstate Medical Center with complaint of intermittent epigastric pain that began a few days prior. The patient was slightly confused at time of presentation but was able to provide some history. Collateral history was collected from his daughter who lives with the patient and was also present at time of the patient's presentation to the ED. The patient reported that his epigastric pain was nonradiating, associated with nausea along with multiple episodes of nonbilious, nonbloody emesis. The patient also reported subjective fevers, chills, and cough for one week. He denied any flank pain, changes in bowel habits, appetite, or amount of oral intake. He also denied any changes in his weight. His daughter, however, noted that over the past week, she observed a decrease in the patient's appetite and oral intake. Patient's medical history was significant for hypertension, for which he did not take any medication, and hepatitis B (Hep B), for which he was hospitalized in 1983. He was unable to recall the cause of the hepatitis. He denied any history of tobacco, alcohol, illicit drug, or recent NSAID use. He does not take any prescribed or over-the-counter medications. His family history was unremarkable.

A thorough occupational history collected later in the course of hospitalization revealed that the patient has worked in the aviation industry as an aircraft refueler at a local airport, where he refuels up to nine aircrafts per day for the past three and half years. At work, the patient reports wearing a uniform and “long medical gloves,” which he described as being approximately five times the thickness of standard hospital gloves. He also stated that he wears a respirator mask about 60% of the time during refueling. The patient reported direct skin contact with jet fuel when it spills onto his arms at least three times per day on average, despite wearing a uniform and gloves. He mentioned one instance, where he was completely covered in jet fuel. He also noted that he could often smell fuel on his hands for several days after a spill. At time of admission, the patient was oliguric and in acute renal failure (ARF) as his serum chemistry showed a strikingly elevated blood urea nitrogen (BUN) and creatinine that were each about 20 times over the normal limits. He was normotensive, and his physical exam was only revealing of dry mucous membranes, clear lungs, and a cardiac exam that was within normal limits. He had no edema of his lower extremities bilaterally. The cause of his ARF was unknown at this time.

The patient's hospital course included extensive medical and diagnostic testing to investigate the etiology of his acute renal failure. His baseline renal function was unknown. The patient's initial serum chemistry showed a BUN level of 215 mg/dL and creatinine level of 20.41 mg/dL with an estimated glomerular filtration rate (eGFR) of 3 mL/min/1.73 m^2^. Electrolytes showed slight hyponatremia with a serum sodium level of 126 mmol/L, normal serum potassium level of 4.6 mmol/L, blood glucose level of 130 mg/dL, normal magnesium level of 2.3 mg/dL, modest hyperphosphatemia level of 5.3 mg/dL, calcium level of 9.1 mg/dL, and a mild metabolic acidosis with a serum bicarbonate level of 17 mmol/L. The patient was seen by the Nephrology consultation service who recommended continuing aggressive intravenous fluid (IVF) hydration and strict urine output monitoring and deferred on initiating renal replacement therapy at the time. The patient had placement of an indwelling Foley catheter for strict urine output measurement. His urinalysis was mainly unrevealing showing no glucosuria, no proteinuria, 11 white blood cells, 6 red blood cells per high-power field, and a specific gravity of 1.010. His calculated fractional extraction of sodium (FeNa) was 6% indicating an intrinsic renal or postobstructive cause. As we continued aggressive IVF hydration, patient's urine output improved and so did his mental status. He became more awake and alert and was able to provide better history. Other workups during hospital course included a negative HIV and negative hepatitis C serology. He had a known history of hepatitis B, and his serology demonstrated prior exposure with developed immunity (positive Hep B core antibody, positive Hep B surface antibody, and a negative Hep B surface antigen). The patient also had normal C3 and C4 levels. C-ANCA and P-ANCA testings were also negative. The creatinine phosphokinase (CPK) level was only modestly elevated to 493 u/L, which is insufficient to explain the degree of renal impairment in the patient. As we continued aggressive hydration, his renal function continued to improve with a quicker fall in the serum creatinine compared to the BUN. Other laboratory testings included an elevated iPTH level of 275.4 pg/mL. The patient also underwent a computed tomography (CAT) scan of the abdomen and pelvis without intravenous contrast that showed no acute pathology in the abdomen, and a renal ultrasound did not show any evidence of obstruction bilaterally but, however, did report mildly echogenic kidneys consistent with medical renal disease. After 11 days of hospitalization, the patient had fully returned to his baseline mental status and was stable for discharge to home. His chemistry on the final day of hospitalization showed a BUN level of 39, serum creatinine level of 1.8 mg/dL, and serum bicarbonate level of 23 mmol/L.

## 3. Discussion

**Figure 1:**
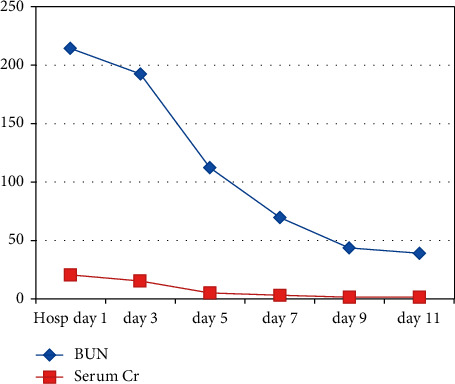
Table showing relationship between patient's renal functional markers (BUN/Cr in mg/dL) over days during the course of hospitalization.

Although the patient's renal function had not returned to normal limits at time of discharge, there was substantial improvement in his serum BUN and Cr from time of admission in comparison to the day of discharge as shown in Figure 1. There was also marked improvement in his clinical and mental status throughout his hospitalization. It is very likely that our patient has underlying chronic kidney disease stage II or III and the BUN/Cr values at discharge are his baseline renal function. His baseline chronic kidney disease diagnosis is suggested by his elevated iPTH and a normal serum calcium, indicative of some bone mineral disease often seen with chronic kidney disease. His renal ultrasounds also showed evidence of underlying medical renal disease, which supports this notion. The patient's baseline chronic kidney disease is likely the result of his untreated longstanding hypertension. We believe that an acute insult led to his ARF and uremia. Through extensive diagnostic testing and careful history taking, we concluded that it is most likely the patient's occupational exposure to jet fuel is implicated in causing his acute nephropathy. Fortunately, we were able to reverse the majority of the damage with aggressive intravenous and then oral fluid hydration. A kidney biopsy would have been helpful in establishing the exact renal pathology and diagnosis but was not pursued in this case because of resolution of the acute renal failure.

To conclude, the assessment of a patient with acute kidney injury should involve a comprehensive history, including an inquiry of potential environmental or occupational exposures. History of either potential toxin exposure or suggestion of Mesoamerican nephropathy may redirect management of kidney injury from other potential pathologies. Further investigation is necessary to establish a relationship between jet fuels and kidney injury.
